# rMVP: A Memory-efficient, Visualization-enhanced, and Parallel-accelerated Tool for Genome-wide Association Study

**DOI:** 10.1016/j.gpb.2020.10.007

**Published:** 2021-03-02

**Authors:** Lilin Yin, Haohao Zhang, Zhenshuang Tang, Jingya Xu, Dong Yin, Zhiwu Zhang, Xiaohui Yuan, Mengjin Zhu, Shuhong Zhao, Xinyun Li, Xiaolei Liu

**Affiliations:** 1 Key Laboratory of Agricultural Animal Genetics, Breeding and Reproduction, Ministry of Education & College of Animal Science and Technology, Huazhong Agricultural University, Wuhan 430070, China; 2 Key Laboratory of Swine Genetics and Breeding, Ministry of Agriculture, Huazhong Agricultural University, Wuhan 430070, China; 3 School of Computer Science and Technology, Wuhan University of Technology, Wuhan 430070, China; 4 Department of Crop and Soil Sciences, Washington State University, Pullman, WA 99164, USA

**Keywords:** Memory-efficient, Visualization-enhanced, Parallel-accelerated, rMVP, GWAS

## Abstract

Along with the development of high-throughput sequencing technologies, both sample size and SNP number are increasing rapidly in genome-wide association studies (GWAS), and the associated computation is more challenging than ever. Here, we present a memory-efficient, visualization-enhanced, and parallel-accelerated R package called “rMVP” to address the need for improved GWAS computation. rMVP can 1) effectively process large GWAS data, 2) rapidly evaluate population structure, 3) efficiently estimate variance components by Efficient Mixed-Model Association eXpedited (EMMAX), Factored Spectrally Transformed Linear Mixed Models (FaST-LMM), and Haseman-Elston (HE) regression algorithms, 4) implement parallel-accelerated association tests of markers using general linear model (GLM), mixed linear model (MLM), and fixed and random model circulating probability unification (FarmCPU) methods, 5) compute fast with a globally efficient design in the GWAS processes, and 6) generate various visualizations of GWAS-related information. Accelerated by block matrix multiplication strategy and multiple threads, the association test methods embedded in rMVP are significantly faster than PLINK, GEMMA, and FarmCPU_pkg. rMVP is freely available at https://github.com/xiaolei-lab/rMVP.

## Introduction

The computation burden of genome-wide association studies (GWAS) is partially caused by the increasing sample size and marker density applied for these studies. As a result, how to efficiently analyze the big data is a big challenge. Additionally, GWAS have been widely used for detecting candidate genes that control human diseases and agricultural economic traits, where the accuracy of the results is of significant implication. Thus, how to achieve higher statistical power under a reasonable level of type I error is another challenge [1]. To efficiently detect more candidate genes with lower false positive rates is the current working goal for GWAS algorithms and tools [2], [3].

Introducing the population structure concept into GWAS has dramatically improved the accuracy of detection. For example, incorporating the fractions of individuals belonging to subpopulations, namely Q matrix, reduces both false positive and false negative signals [4]. Principal components (PCs) are widely used to represent subpopulations and to enable the incorporation of population structure into GWAS [5]. Implementing the general linear model (GLM) to incorporate either the Q matrix or PCs as covariates, PLINK has become the most popular software package for GWAS [6].

False positives also stem from individuals that exhibit high variability in pairwise relatedness presumptively classified into different subpopulations. In addition to integrating population structure, statistical power can be substantially improved by the incorporation of hidden relationships in a mixed linear model (MLM) — particularly when population structure is less dominant than the cryptic relatedness [7]. Multiple algorithms have been developed to boost both the computational efficiency and statistical power of MLM methods [8], [9], [10], [11]. Various software packages have also been developed for the implementation of these algorithms, including TASSEL [12], GAPIT [13], [14], GenABEL [15], Efficient Mixed-Model Association eXpedited (EMMAX) [16], GEMMA [17], and GCTA [18]. Even though the number of GWAS literatures applying MLM-based methods is increasing rapidly, the applications of the MLM-based software are still not comparable with that of PLINK software, primarily because PLINK operates much faster than MLM-based software.

Besides the difference in computing time, MLM does not provide high statistical power compared to GLM. The difference in statistical power between GLM and MLM is negligible in some scenarios, such as mapping genes under the same false discovery rate (FDR) in populations with strong population structure [19]. These populations include human populations, as well as animal and plant populations that have been isolated by breeding programs. Our newly developed method, fixed and random model circulating probability unification (FarmCPU), has higher statistical power than both GLM and MLM for evaluating populations with either weak or strong population structure. FarmCPU splits MLM into a fixed effect model (FEM) and a random effect model (REM), using them iteratively to increase the power for detecting candidate genes associated with population structure. Association tests in FarmCPU are validated by FEM with the same computing efficiency as GLM, while the statistical power surpasses that of MLM at the same level of type I error.

Although recently developed methods have improved statistical power under certain assumptions, determining the most appropriate method for a given dataset is still convoluted. Human genetic studies often use large datasets with simple models, while plant and animal genetic studies prefer complex models with limited sample sizes. For a specific trait, it is usually difficult to identify the real genetic architecture and the most appropriate method to be used. Researchers have to try out multiple methods and identify candidate genes based on both statistical and biological evidence. Additionally, existing GWAS software rarely focus on providing a flexible plotting function to display GWAS-related information in a way that satisfies the personal aesthetic requirements of the researchers. Furthermore, with the development of multi-trait methods, such as GSA-SNP2 [20], MTMM [21], mvLMMs [22], and mtSet [23], results from multiple-group GWAS need to be displayed simultaneously for easier comparisons. Therefore, there appears a need for analyzing big data with limited computing memory, reasonable time, and reduced false positive rates, while displaying the results in high-quality figures. To address all of the aforementioned requirements, we developed the memory-efficient, visualization-enhanced, and parallel-accelerated package (rMVP) in R.

## Method

We split the entire GWAS procedure into six sections: data preparation, evaluation of population structure, estimation of variance components, association tests, globally efficient design on GWAS process computing, and result visualization. Abundant functions have been implemented in rMVP for each section.

### Data preparation

rMVP accepts multiple popular formats for genotype files, such as PLINK binary, Hapmap, VCF, and Numeric [*e.g.*, genotype data coded as integer (0, 1, 2) or dosage/probability (0.1, 0.3, 0.6)]. All aforementioned formats will be converted to the ‘big.matrix’ format. The advantage of converting genotype files into ‘big.matrix’ is that the size of the file is only limited by the storage capacity of the hard disk but not the processing capacity of random access memory (RAM; ‘memory’ is referred to RAM in this manuscript) [24].

### Evaluation of population structure and individual relationship

For population structure analysis, PCs can be calculated using all available markers. An ideal population for GWAS assumes that the individuals are randomly selected from a big population, but the population could always be classified to multiple subpopulations in fact. As the alleles with different frequencies in different subpopulations would generate false positives, we recommend to integrate the top 3–5 top PCs as covariates into model to control false positives caused by population structure following previous studies [5], [19]. VanRaden method is implemented in rMVP for the efficient construction of genomic relationship matrix (GRM) [25].

### Estimation of variance components

Four algorithms are implemented for estimating variance components in rMVP: Brent (default method in rMVP) [26], EMMAX / Population Parameters Previously Determined (P3D) [8], [16], Factored Spectrally Transformed Linear Mixed Models (FaST-LMM) [9], and Haseman-Elston (HE) regression [27]. Different algorithms have different performances in terms of accuracy and efficiency. For instance, Brent and EMMAX use Eigen decomposition on GRM to avoid computing the inverse of big matrix; FaST-LMM use singular value decomposition (SVD) on genotype matrix, which can be more efficient when the number of markers is far less than the number of individuals; HE regression, which uses the linear regression model to fit the similarity of phenotype and GRM among individuals, is less accurate but can be much more memory-efficient and time-saving, making it more promising in very large datasets.

### Association tests

GLM, MLM, and FarmCPU methods are implemented in rMVP for association tests. When there are more than one covariate (*e.g.*, PCs) added to association test models, the inverse of the design matrix corresponding to the covariates will be calculated *n* times, where *n* is marker size. Block matrix multiplication strategy can be used to speed up the processes including inverse of the design matrix corresponding to the covariates and the testing markers. This strategy is used in all available association test methods in rMVP. Take GLM as an example, the FEM function can be written as:

(1) y=Xb+e

where y is a vector of phenotype, X is a matrix of fixed effects and testing SNPs, b is an incidence matrix for X, and e is a vector of residual that follows a normal distribution with mean of zero and $I\sigma_{e}^{2}$ covariance, where I is the identity matrix and $\sigma_{e}^{2}$ is the unknown residual variance. Equation (1) can be reformulated by following steps:

$$X^{\text{'}}y=X^{\text{'}}Xb$$

(2) $$b={\left(X^{\text{'}}X\right)}^{-1}X^{\text{'}}y$$

where $X^{\text{'}}$ is the transpose matrix of X. If there are *k* fixed effect vectors added as covariates in the model, X and b can be written as:

$$X=[C_{1}^{\text{'}},C_{2}^{\text{'}},C_{3}^{\text{'}},\cdots ,C_{k}^{\text{'}},{\mathrm{SNP}}^{\text{'}}]$$

$$b=[b_{1},b_{2},b_{3},\cdots ,b_{k},c]$$

where $C_{1},C_{2},C_{3},\cdots ,C_{k}$ represent *k* fixed effect vectors and SNP represents the testing SNP vector. Equation (2) can be written as.

(3) $$\left[\begin{array}{c} b_{1}\\ b_{2}\\ b_{3}\\ \cdots \\ b_{k}\\ c \end{array}\right]={\left(\left[\begin{array}{c} C_{1}\\ C_{2}\\ C_{3}\\ \cdots \\ C_{k}\\ SNP \end{array}\right]\left[C_{1}^{\text{'}},C_{2}^{\text{'}},C_{3}^{\text{'}},\cdots ,C_{k}^{\text{'}},{\mathrm{SNP}}^{\text{'}}\right]\right)}^{-1}\left[\begin{array}{c} C_{1}\\ C_{2}\\ C_{3}\\ \cdots \\ C_{k}\\ SNP \end{array}\right]y$$

The most time-consuming part in Equation (3) is the inverse of M matrix. And M is defined as:

$$M=\left[\begin{array}{c} C_{1}\\ C_{2}\\ C_{3}\\ \cdots \\ C_{k}\\ SNP \end{array}\right]\left[C_{1}^{\text{'}},C_{2}^{\text{'}},C_{3}^{\text{'}},\cdots ,C_{k}^{\text{'}},{\mathrm{SNP}}^{\text{'}}\right]$$

If we use w and x represent $C_{1},C_{2},C_{3},\cdots ,C_{k}$ and SNP, respectively, the inverse of M matrix can be written as:

$$M^{-1}={\left(\left[\begin{array}{c} w^{\text{'}}\\ x^{\text{'}} \end{array}\right]\left[w,x\right]\right)}^{-1}={\left[\begin{array}{cc} w^{\text{'}}w & w^{\text{'}}x\\ x^{\text{'}}w & x^{\text{'}}x \end{array}\right]}^{-1}=\left[\begin{array}{cc} M_{11} & M_{12}\\ M_{21} & M_{22} \end{array}\right]$$

where

$$M_{11}={\left(w^{\prime }w\right)}^{-1}+{\left(w^{\prime }w\right)}^{-1}w^{\prime }x{\left(x^{\prime }x-x^{\prime }w{\left(w^{\prime }w\right)}^{-1}w^{\prime }x\right)}^{-1}x^{\prime }w{\left(w^{\prime }w\right)}^{-1}$$

$$M_{12}=-{\left(w^{\prime }w\right)}^{-1}w^{\prime }x{\left(x^{\prime }x-x^{\prime }w{\left(w^{\prime }w\right)}^{-1}w^{\prime }x\right)}^{-1}$$

$$M_{21}=-{\left(x^{\prime }x-x^{\prime }w{\left(w^{\prime }w\right)}^{-1}w^{\prime }x\right)}^{-1}x^{\prime }w{\left(w^{\prime }w\right)}^{-1}$$

$$M_{22}={\left(x^{\prime }x-x^{\prime }w{\left(w^{\prime }w\right)}^{-1}w^{\prime }x\right)}^{-1}$$

The inversion of $w^{\prime }w$ matrix is recomputed *n* times when constructing $M_{11},M_{12},M_{21},M_{22}$ matrix for each testing marker. For the matrix operations in GLM, MLM, and each iteration of FarmCPU, the w matrix is fixed, and the inversion of $w^{\prime }w$ can be calculated only once using block matrix multiplication strategy. As it is repeated *n* times when testing the SNPs, more time will be saved when there are more covariates in the model or more SNPs to be tested.

### Globally efficient design of GWAS calculations

A standard GWAS pipeline generally includes PC derivation, GRM construction, variance component estimation, and association tests. There are three commonly used strategies for deriving the PCs. 1) The Eigen decomposition results of the matrix that represents the correlation coefficients between pairs of markers could be derived by $\left(M^{T}M\right)v=\lambda v$, where M is a *n* by *m* scaled genotype matrix (*n* is the number of individuals, and *m* is the number of SNPs). The Eigen decomposition analysis is conducted on the correlation matrix $M^{T}M$, the dimension of which is *m* by *m*, and this would lead to high requirements of both memory and computing time with the increasing number of SNPs. 2) The SVD analysis could be conducted on the M matrix by $M=U\Sigma V^{*}$. Its computational complexity is relative smaller than the method that described in 1), as it only needs to decompose a *n* by *m* matrix. 3) The PCs could be also derived by performing the Eigen decomposition of the GRM, which could be calculated by $\mathrm{GRM}=M^{T}M/m$, and its dimension is *n* by *n*. In the majority of cases, the number of markers (*m*) is far more than the number of individuals (*n*), and thus this method has the smallest computational complexity compared with the other two. Moreover, the construction of GRM is always a key part in a commonly used GWAS procedure, which has been precomputed already. Not only that, as shown in Figure S1, the Eigen decomposition results of GRM could be easily applied to processes of variance component estimation and association tests. By the default sets in rMVP, the Eigen decomposition analysis was conducted on GRM, which was constructed by VanRaden method [25]. The methodological formula of VanRaden method can be defined as:

(4) $$G=Z^{T}Z/\sum_{i=1}^{n}p_{i}\left(1-p_{i}\right)$$

where Z is the dimension of a *m* by *n* matrix (*m* is the number of markers, and *n* is the number of individuals), which can be derived from cantering the additive genotype matrix that was coded as 0, 1, and 2 for genotypes AA, AB, and BB, respectively; p is the minor allele frequency. After the Eigen decomposition was finished, the Eigen values and Eigen vectors could be applied to the variance component estimation using Brent method [26], which has fast convergence determined via the absolute tolerance of heritability rather than all variance components; and the results of Eigen decomposition could be also used for solving the mixed model equation when MLM is selected for the association tests. The globally efficient calculation design of GWAS process makes rMVP only need to do the Eigen decomposition once instead of doing it multiple times. Moreover, the results of Eigen decomposition could be directly used in calculation of PC derivation, variance component estimation, and association tests, and thus the computing time is greatly decreased.

### Visualization of results

High-quality figures are generated to display data information, population structure, and GWAS results, including marker density plot, phenotype distribution plot, principal component analysis (PCA) plot, Manhattan plot, and Quantile-Quantile (Q-Q) plot.

## Results

### Memory-efficient: efficient memory usage in data loading and parallel computation

Genotype matrices are the biggest datasets for GWAS. In rMVP, genotype data in multiple formats are converted to ‘big.matrix’, which can minimize RAM usage through generating a bridge that facilitates RAM accessing the data on the hard disk instead of loading it to RAM directly as the most software tools do. rMVP achieves this goal by using the ‘bigmemory’ package to build data mirrors that are accessible to RAM, while the actual data remain on the hard drive. In this way, very little RAM capacity is needed for the temporary storage of the data. Once the data mirrors are built, users will never need to re-build them again and the time of loading input data is negligible. When multiple threads are used to accelerate the association tests, no additional data mirrors will be copied for each thread as all threads will share the same data mirrors.

**Figure 1:**
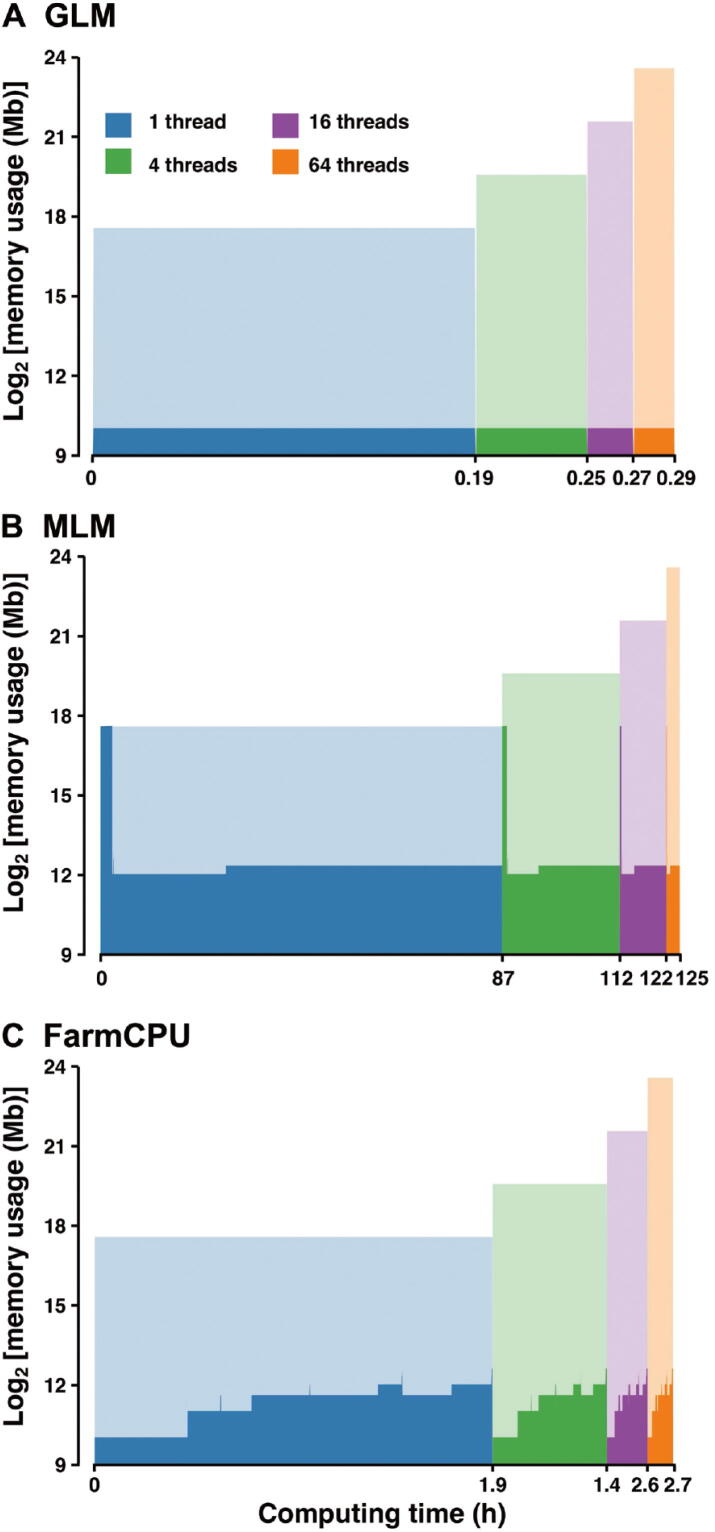
**Comparison of memory usage in response to number of threads used for parallel computation under “speed” mode of rMVP** For each block with a specific color, the y-axis represents memory usage (Mb) in log_2_ scale; the x-axis represents computing time (h). Different color represents different number of threads used for parallel computation. The height of area in dark color represents real memory costs, while the height of shadow in light color represents theoretical memory costs which are 1, 4, 16, and 64 times of genotype data size in standard R matrix format under ‘fork’ parallel mode, respectively. Data for speed test are generated by PLINK software, and each data unit represents 1000 samples and 100,000 SNPs. The data size for testing memory usage is 16 data units (16,000 samples and 1,600,000 SNPs), and 10 PCs are added as covariates in all test methods. All tests are performed on a Red Hat Enterprise Linux sever with 2.60 GHz Intel(R) Xeon(R) 32CPUs E5-4620 v2 and 512 GB memory. GLM, general linear model; MLM, mixed linear model; FarmCPU, fixed and random model circulating probability unification; PC, principal component.

Here, we made a rough illustration of ‘big.matrix’-based memory storage of one and multiple threads for rMVP. The complete GWAS procedure of three methods was recorded for RAM usage test in a Linux server (‘RES’–‘SHR’). In this test, the product of genotype data size was measured in standard R matrix format, and ‘theoretical RAM cost’ for multiple threads in ‘fork’ mode is defined as *r* × *c* × *t* × 8 bytes, where *r* and *c* are the number of rows and columns of a matrix, respectively, and *t* is the number of threads. From the results shown in Figure 1, we concluded that, with more threads, rMVP shares variables in RAM among processers and but does not require additional memory compared with single thread by the aid of Open Multi-Processing (OpenMP) parallel acceleration. Moreover, by constructing memory-map file for genotype in disk rather than load it all into RAM, rMVP significantly decrease the memory cost, making rMVP pretty promising in processing big data at a personal computer with limited computing resources.

For MLM in Figure 1, a high shoulder peak appears at the beginning of the memory records, indicating that the most memory cost part of the MLM is the construction of GRM. From the computation details of VanRaden method described above (Equation (4)), we can conclude that the calculation of $Z^{T}Z$ requires gigantic storage space and the requirement is increasing with both the marker size and the number of individuals. To take care of this problem, we implement two modes (“speed” and “memory”) in rMVP to handle the big data with limited computation resources.

For the “speed” mode, the genotype matrix is stored in the standard R matrix format and the transpose of Z matrix and the matrix multiplication are carried out by the RcppArmadillo package, which could be automatically speeded up by the Inter Math Kernel Library (MKL) based on Microsoft R Open platform. However, the big genotype data are loaded into RAM, resulting in a big memory cost as most of the GWAS software tools do (*e.g.*, GEMMA, GCTA, and GAPIT). For the “memory” mode, all the matrices that are required for constructing the GRM are stored in the ‘big.matrix’ format and the matrix multiplication of ‘big.matrix’ is implemented by our newly developed C++ function, which could be parallel accelerated by using the OpenMP technology. Although it can significantly decrease the memory cost, more computing time is required (Table 1). Users can easily adjust the “priority” parameter to get rid of the memory limit or obtain the fastest speed depending on the data size and computing resources.

**Table 1:** **Comparison of memory and time costs between the “speed” and “memory” modes in rMVP**

Mode	Data unit (memory/time, Gb/min)				
	1	2	4	8	16
Speed	0.51/0.05	3.28/0.15	17.80/0.6	73.10/3.2	285.60/34.70
Memory	0.06/0.20	0.08/1.61	0.17/9	0.53/42.12	2.06/461.66

*Note*: Data for speed test are generated by PLINK software and each data unit represents 1000 samples and 100,000 SNPs. Parallel computation with 32 CPUs is used to speed up for both modes. All tests are performed on a Red Hat Enterprise Linux sever with 2.60 GHz Intel(R) Xeon(R) 32CPUs E5-4620 v2 and 512 GB memory.

### Parallel-accelerated: parallel computation and block matrix multiplication for accelerating association tests

#### Speed up by block matrix multiplication

Most GWAS models contain several columns of covariates, such as PCs and Sex, and the linear model function has to be solved for every single testing marker. This process involves the inverse of the design matrix for covariates and testing markers. Since the covariates are the same for every testing marker, we partitioned the design matrix into sub-matrices according to the covariates and the testing markers. The inverse of the entire design matrix was calculated from the one-time calculation of the inverse of the sub-matrix of covariates. As the number of covariates and markers increased, sub-matrix partitioning significantly saved computing time (Table 2). Block matrix multiplication strategy has been used in all association tests including GLM, MLM, and FarmCPU.

**Table 2:** **Speed performance of GLM with and without using block matrix multiplication strategy**

No. of covariates	Time (s)	
	Without using block matrix multiplication strategy	With using block matrix multiplication strategy
0	1012	597
3	2853	614
5	4908	623
10	10,837	681

*Note*: 0, 3, 5, and 10 covariates are added in both PLINK v1.9 and rMVP for testing speed of GLM with and without using block matrix multiplication strategy, respectively. A dataset including 16,000 samples with 1,600,000 SNPs is generated by PLINK software and used for test. All tests are performed using single thread. GLM, general linear model.

#### Speed up by parallel computation

**Figure 2:**
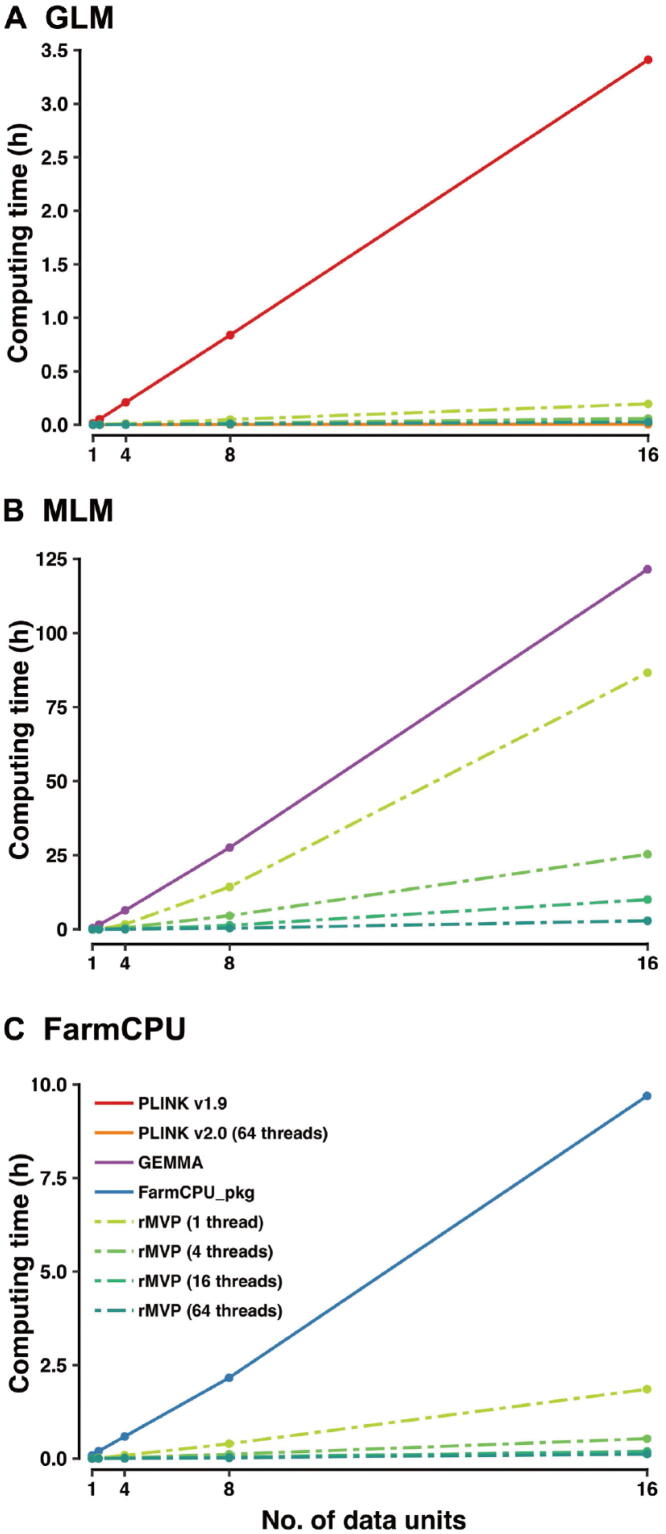
**Comparison of computing speed of PLINK, GEMMA, FarmCPU_pkg, and rMVP (“Speed” mode)** Computing time (h) in response to the number of data units is displayed, and 5 PCs are added as covariates in all test methods. Speed performances of association test methods GLM, MLM, and FarmCPU in rMVP are analyzed using 1, 4, 16, 64 threads, and are compared to the speed performances of relative methods in PLINK, GEMMA, and FarmCPU_pkg, respectively. Data for speed test are generated by PLINK software, and each data unit represents 1000 samples and 100,000 SNPs. The biggest dataset for speed test of all models are 16 data units (16,000 samples and 1,600,000 SNPs). All tests are performed on a Red Hat Enterprise Linux sever with 2.60 GHz Intel(R) Xeon(R) 32CPUs E5-4620 v2 and 512 GB memory.

There are two levels of parallel computation implemented in rMVP: data level parallel (DLP) and thread level parallel (TLP). For DLP, based on the Microsoft R Open platform, multi-threads have been automatically assigned to speed up the mathematical calculation, such as matrix manipulation. For TLP, association tests on millions of markers are allocated to a group of threads and calculated simultaneously. rMVP switches between the two levels of parallel computation to achieve the highest speed based on the biggest computation requirements in different GWAS procedures. Since three association test methods (GLM, MLM, and FarmCPU) in rMVP nearly generated consistent association results (Figure S2) with and same Power/FDR performance (Figure S3) as related methods in PLINK v2.0 (written in C++, https://www.cog-genomics.org/plink/2.0/), GEMMA (written in C++, https://github.com/genetics-statistics/GEMMA/), and FarmCPU_pkg (R package written in pure R, https://zzlab.net/FarmCPU/), respectively, rMVP (written in R and C++) was compared with these software packages for speed performance; the computing time was recorded for each software from loading data to generating result files (Figure 2; Table S1). Detailed software version and scripts used for computing speed test are provided in Table S2.

Benefiting from the block matrix multiplication and parallel computation strategies, rMVP is several times or even dozens of times faster than PLINK (v1.9), GEMMA, and FarmCPU_pkg at single-thread level, and the gap increases significantly for multiple-thread computing.

### Visualization enhanced: flexible adjustments for generating high-quality figures

‘MVP.report’ function provides a pack of high-quality figures for visualizing GWAS-related information, including data information, population structure, and GWAS results.

**Figure 3:**
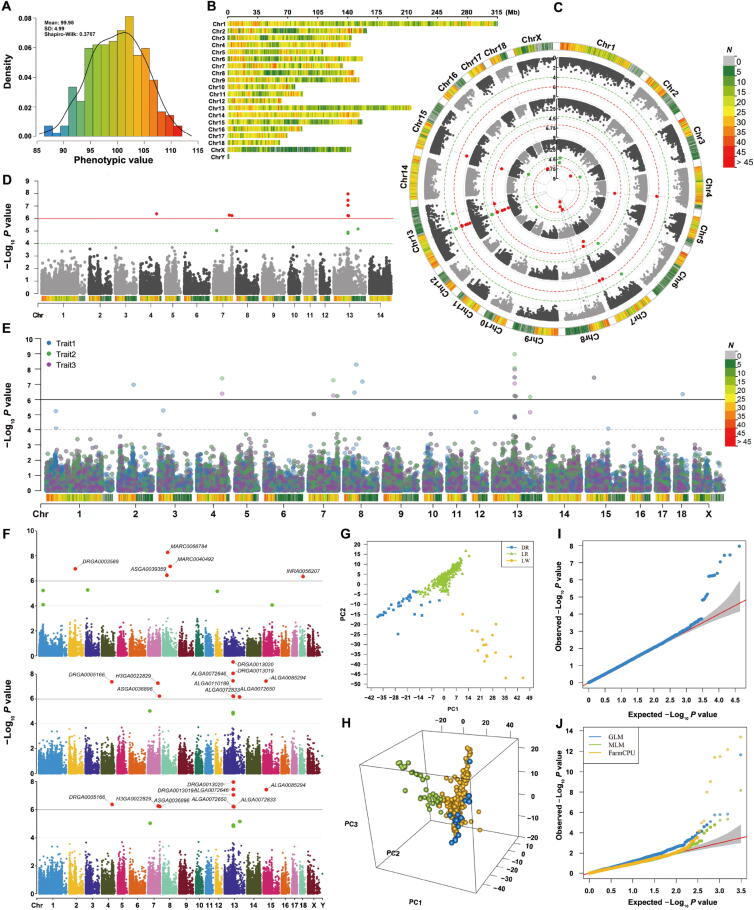
**Visualization of GWAS-related information** **A.** Phenotype distribution. **B.** Marker density showing with color lumps with a user-defined window size (*e.g.*, 1 Mb). **C.** Manhattan plot for multiple-group GWAS results in circular manner. **D.** Manhattan plot for single-group GWAS results with marker density information. **E.** Manhattan plot for multiple-group GWAS results in one set of rectangular axes. **F.** Manhattan plot for multiple-group GWAS results in multiple sets of rectangular axes. **G.** Visualization of population structure in two dimensions. **H.** Visualization of population structure in three dimensions. **I.** Q-Q plot for single-group GWAS results. **J.** Q-Q plot for multiple-group GWAS results.

Visualization of data information includes phenotype distribution (Figure 3**A**) and marker density (Figure 3B), which are used to show if the phenotype is normally distributed and the SNPs are evenly covered the entire genome. Skewed phenotype distribution and uneven distributed genotype data would result false positives and biased estimation of population structure and relationship among individuals.

Besides, rMVP also provides various types of high-quality plots, including Manhattan plot, PCA plot, and Q-Q plot. Marker density information is added to Manhattan plot to show the marker coverage of candidate regions (Figure 3C–E). Multiple-group GWAS results can be visualized on a same Manhattan plot, and users could highlight some SNPs or genes of interest on the Manhattan plot without overlap (Figure 3F). Top PCs are visualized in manner of both two and three dimensions to display the population structure (Figure 3G and H), and Q-Q plots (Figure 3I and J) for both single trait and multiple traits can be output simultaneously for users’ specific requirements. Our ‘MVP.report’ can also easily process GWAS results from other software for visualization, such as PLINK, GEMMA, GCTA, and TASSEL. This function can be further extended to visualize the results from analyses of multi-omics, correlated traits, and expression quantitative trait loci (eQTLs), and to display the commonly detected candidate areas. Users can make desired output figures using more than 40 parameters. Detailed descriptions for all parameters are listed in Table S3 and File S1.

## Discussion

A summary of GWAS-related functions of rMVP compared with other software tools is listed in Table 3. At the moment, rMVP does not provide functions of imputation and quality control, which need to be done before association tests. Instead, rMVP provides functions for flexible data conversion that can easily accept the data from other software, such as Beagle, which also accepts data in VCF format and provides imputation and quality control functions [28].

**Table 3:** **Summary of GWAS-related functions in PLINK, GEMMA, FarmCPU_pkg, and rMVP**

Function	Item	Software			
		PLINK	GEMMA	FarmCPU_pkg	rMVP
Input	Hapmap	×	×	√	√
	VCF	√	×	×	√
	Binary	√	√	×	√
	Numeric	×	×	√	√
	BIMBAM	×	√	×	×
	Quality control	√	×	×	×
Model	GLM	√	√	√	√
	MLM	×	√	×	√
	FarmCPU	×	×	√	√
Population structure	PCs	√	×	√	√
	GRM	×	√	×	√
Variance component estimation	Brent	×	×	×	√
	EMMAX	×	×	√	√
	Fast-LMM	×	×	√	√
	HE regression	×	√	×	√
Output	*P* value, SE, effect	√	√	√	√
	Manhattan plot	×	×	√	√
	Q-Q plot	×	×	√	√
	SNP density plot	×	×	×	√
	Phenotype distribution	×	×	×	√
	PCA plot	×	×	√	√

*Note*: √, software with related functions; ×, software without related functions. MLM, mixed linear model; FarmCPU, fixed and random model circulating probability unification; PC, principal component; GRM, genomic relationship matrix; EMMAX, Efficient Mixed-Model Association eXpedited; FaST-LMM, Factored Spectrally Transformed Linear Mixed Models; HE regression, Haseman-Elston regression; SE, standard error; Q-Q plot, Quantile-Quantile plot; PCA, principal component analysis.

rMVP currently only supports DLP and TLP for parallel computation, lacking the implementation of distributed parallel system (DPS). Compared with TLP that can speed up the computation using 100 threads on a single node, DPS (*e.g.*, MPI, Hadoop, and Spark) can distribute the tasks to 1000 threads on multiple nodes. DPS is also better at dealing with hundreds or thousands of phenotypes and large computing tasks that need to be split, but its performance is limited by the efficiency of data transfer among multi nodes through the local network. However, association tests in rMVP can be accomplished within 10 h for a dataset that includes 500,000 samples and 5,000,000 markers for each sample using FarmCPU model, suggesting that our rMVP can meet most users’ requirements.

Future work includes implementing efficient imputation and quality control functions, and supporting DPS to meet the challenge of big datasets with millions of samples. We also plan to incorporate more association test methods, such as logistic regression and multi-trait model (fitting binary and multi-genetically-correlated traits). With the development of graphic processing unit (GPU) technology, we can get thousands of cores and higher memory bandwidth at a low price. Most of the processes in the GWAS analysis have good independence and can give full play to the advantages of GPU parallel computing. However, the bottleneck of limited GPU memory makes it difficult to perform GPU-based GWAS analysis on a large population. In the future, we plan to extend rMVP to support parallel computing on multiple machines with each machine containing multiple GPUs, and explore new memory optimization methods. Incorporating the aforementioned methods will greatly improve the versatility of rMVP.

## Code availability

The rMVP package is available on both CRAN (https://cran.r-project.org/web/packages/rMVP) and GitHub (https://github.com/xiaolei-lab/rMVP).

## Competing interests

The authors have declared no competing interests.

### CRediT authorship contribution statement

**Lilin Yin:** Data curation, Writing – original draft, Visualization, Software. **Haohao Zhang:** Writing – original draft, Software. **Zhenshuang Tang:** Validation. **Jingya Xu:** Validation. **Dong Yin:** Validation. **Zhiwu Zhang:** Software. **Xiaohui Yuan:** Resources, Writing – review & editing. **Mengjin Zhu:** Writing – review & editing. **Shuhong Zhao:** Writing – review & editing. **Xinyun Li:** Supervision, Writing – review & editing. **Xiaolei Liu:** Supervision, Methodology, Software, Writing – original draft.

## Supplementary material

Supplementary File S1 **Demo scripts and figures for visualization in rMVP**.

Supplementary Figure S1 **The road mapping of whole GWAS procedures in rMVP**. K is the Kinship matrix, also known as GRM. EigenK represents the Eigen decomposition of GRM. GRM, genomic relationship matrix; PC, principal component. VC, variance component.

Supplementary Figure S2 **Comparison of association results for three GWAS models between rMVP and related software**. The experiment was performed on the simulated 16 data units (16,000 samples and 1,600,000 SNPs). The x-axis represents the computed P value in −Log10 format of rMVP for three GWAS models, while the y-axis represents the computed P value in −Log10 format of related software for corresponding GWAS model.

Supplementary Figure S3 **Comparison of power and FDR for three GWAS models between rMVP and related software**. The experiment was performed using an Arabidopsis dataset, which includes 1178 individuals and 208,794 SNPs. The phenotype was simulated by randomly selected 10 quantitative trait nucleotides (QTNs) following a normal distribution with mean = 0 and variance = 0.1, and the heritability was 0.5. The final results were the average of 100 replicates. FDR, false discovery rate.
