# Supplementary material for: rMVP: A Memory-efficient, Visualization-enhanced, and Parallel-accelerated Tool for Genome-wide Association Study
Source: Genomics Proteomics Bioinformatics. 2021 Mar 2;19(4):619–28. doi: 10.1016/j.gpb.2020.10.007 (PMC9040015; doi:10.1016/j.gpb.2020.10.007)

**Demo scripts and figures for visualization in rMVP**

- 1. **Visualization of data information**

1. **Phenotype distribution**

***MVP.Hist****(phe=phenotype, file="jpg", breakNum=18, dpi=300)*


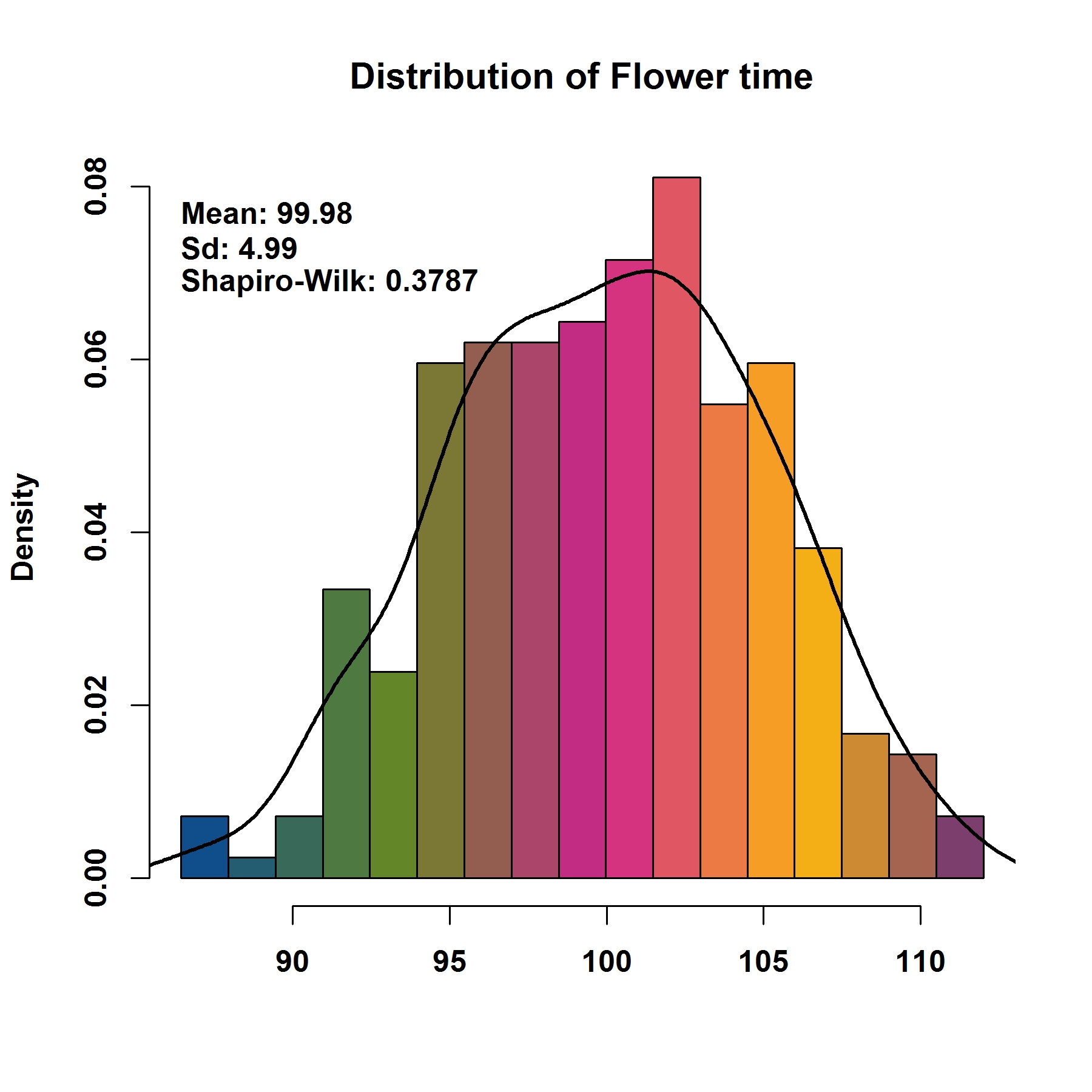


1. **Marker density**

***MVP.Report****(pig60K[, c(1:3)], plot.type="d", col=c("darkgreen", "yellow", "red"), file="jpg", dpi=300)*


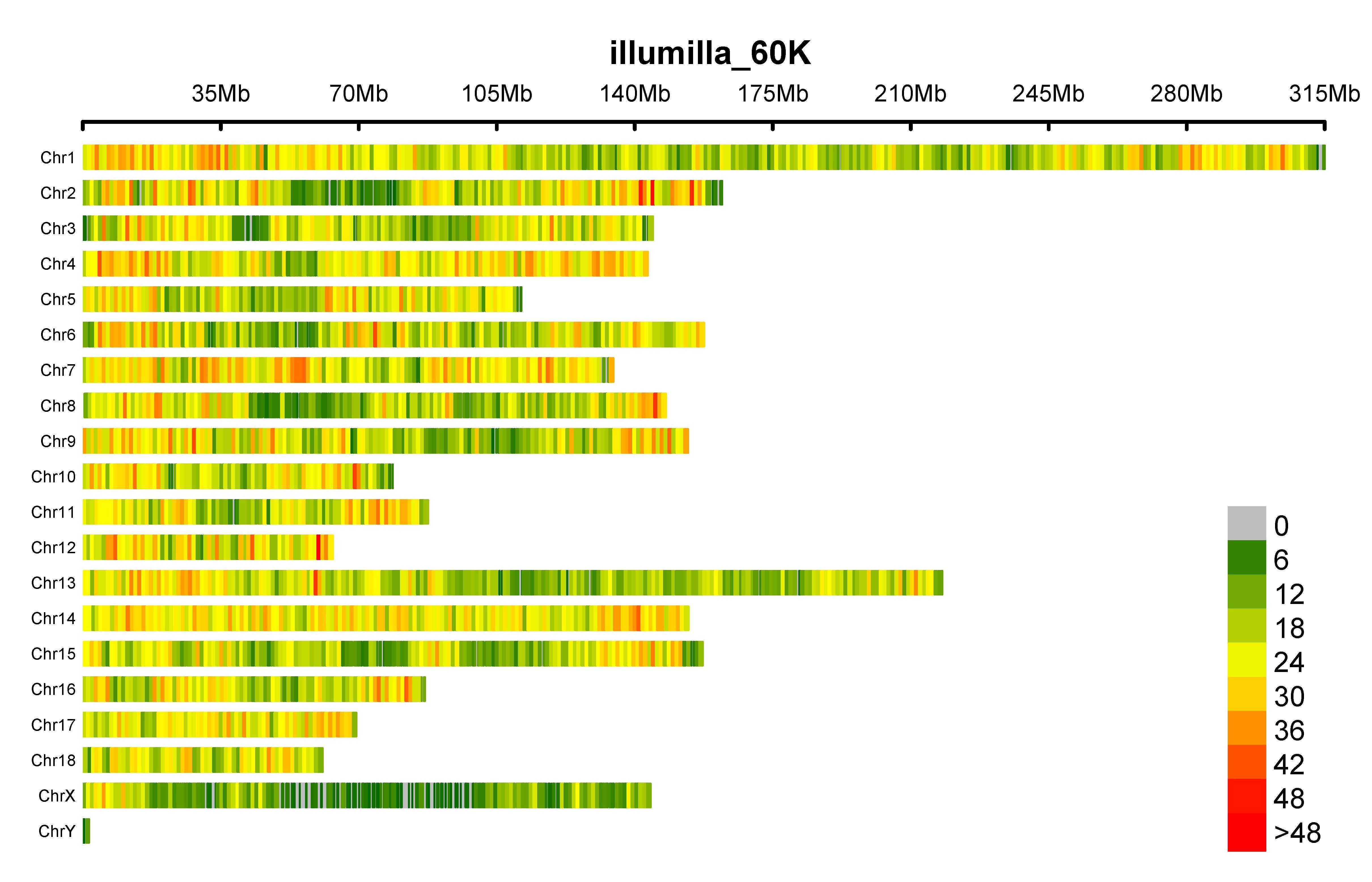


1. **Population structure**

*pca <- attach.big.matrix("mvp.pc.desc")[, 1:3]*

***MVP.PCAplot****(PCA=pca, Ncluster=3, class=NULL, col=c("red", "green", "yellow"), file="jpg", plot3D=TRUE, pch=19)*


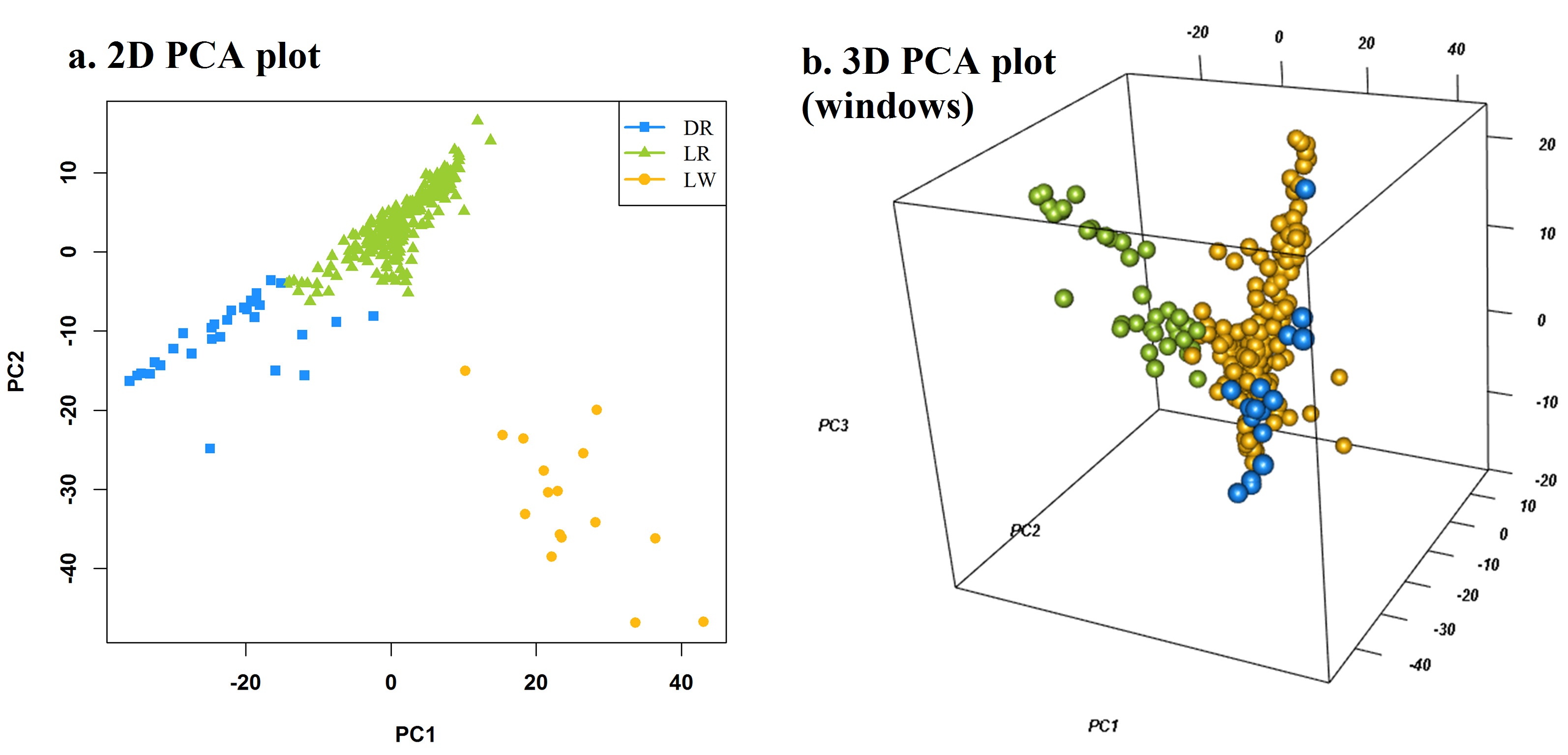


- 1. **Visualization of GWAS results**

1. **Manhattan plot for single-group GWAS result**

***MVP.Report****(pig60K, plot.type="m", LOG10=TRUE, ylim=NULL, threshold=c(1e-6,1e-4), threshold.lty=c(1,2), col=c("grey60","grey30"), threshold.lwd=c(1,1), threshold.col=c("black","grey"), amplify=TRUE, chr.den.col=c("darkgreen", "yellow", "red"), bin.size=1e6, signal.col=c("red","green"), signal.cex=c(1,1), signal.pch=c(19,19), file="jpg", memo="", dpi=300)*


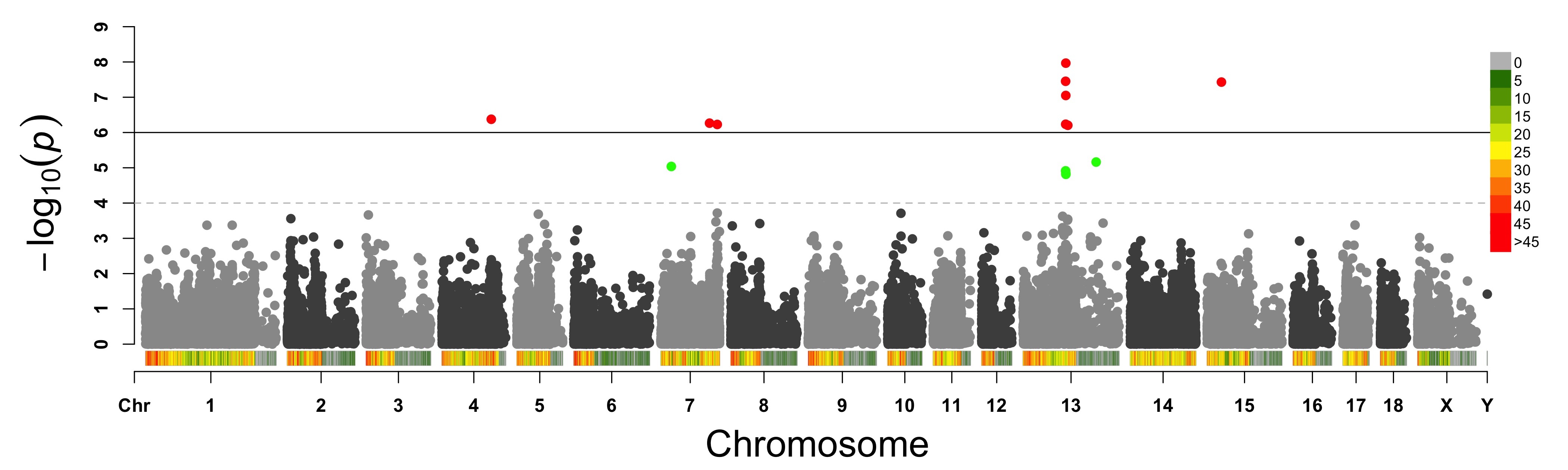


***MVP.Report****(cattle50K, plot.type="m", band=0, LOG10=FALSE, ylab="Abs(SNP effect)", threshold=0.015, threshold.lty=2, threshold.lwd=1, threshold.col="red", amplify=TRUE, signal.col=NULL, col=c("dodgerblue4","deepskyblue"), chr.den.col=NULL, file="jpg", memo="", dpi=300)*


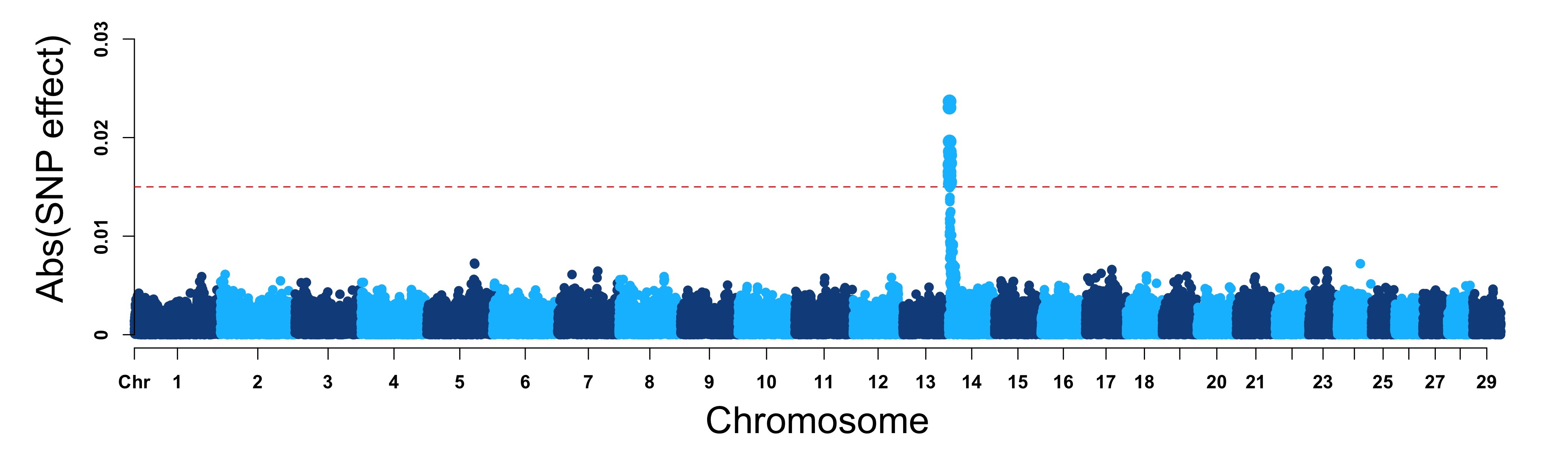


1. **QQ-plot for single-group GWAS result**

***MVP.Report****(pig60K,plot.type="q",conf.int.col=NULL, box=TRUE, file="jpg", memo="", dpi=300)*


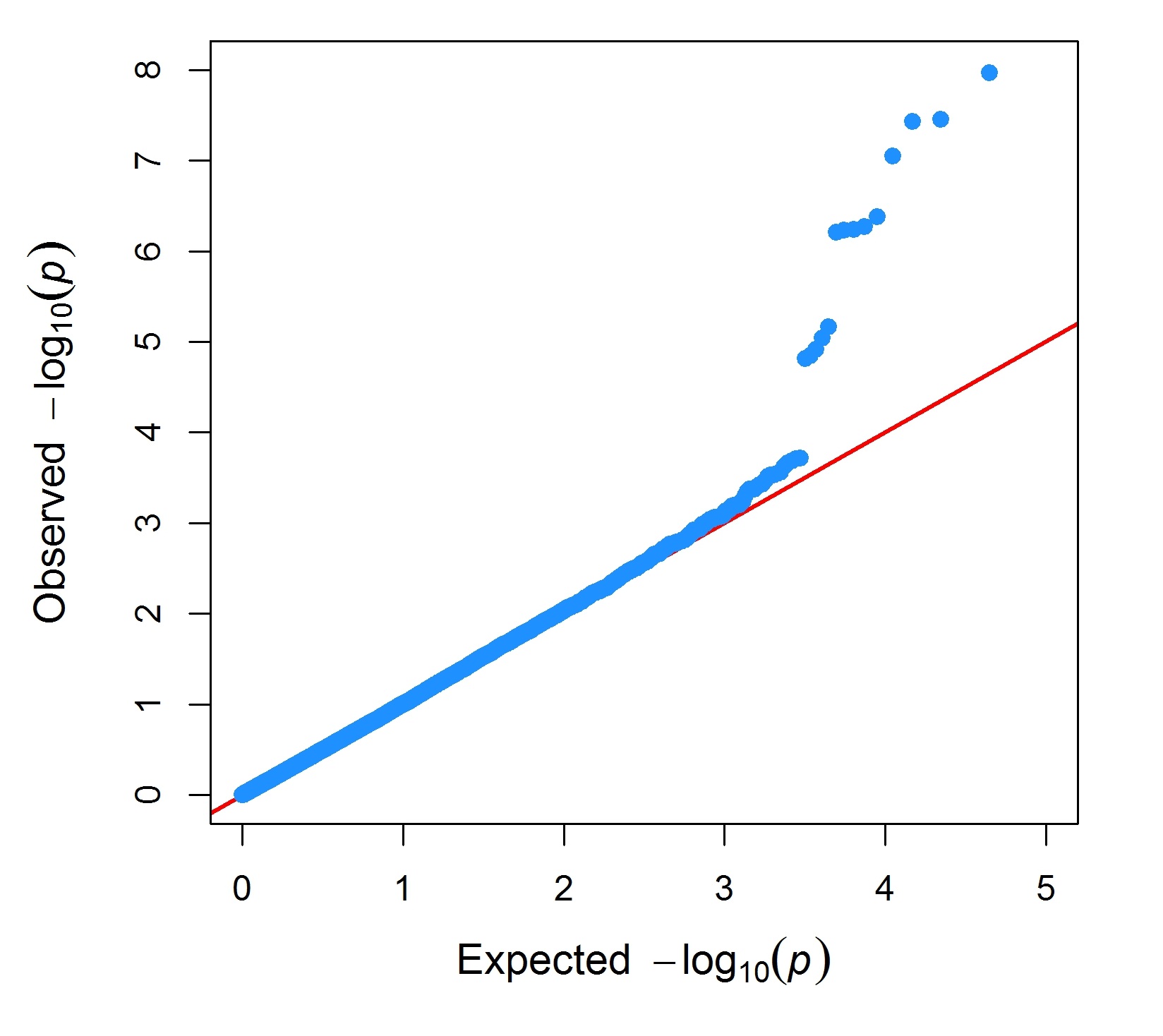


1. **Manhattan plot in circular manner for multiple-group GWAS results**

***MVP.Report****(pig60K, plot.type="c", r=0.4, col=c("grey30","grey60"), chr.labels=paste("Chr",c(1:18,"X"),sep=""), threshold=c(1e-6,1e-4), cir.chr.h=1.5, amplify=TRUE, threshold.lty=c(1,2), threshold.col=c("red","blue"), signal.line=1, signal.col=c("red","green"), chr.den.col=c("darkgreen","yellow","red"), bin.size=1e6, outward=FALSE, bin.size=1e6, file="jpg", memo="", dpi=300)*


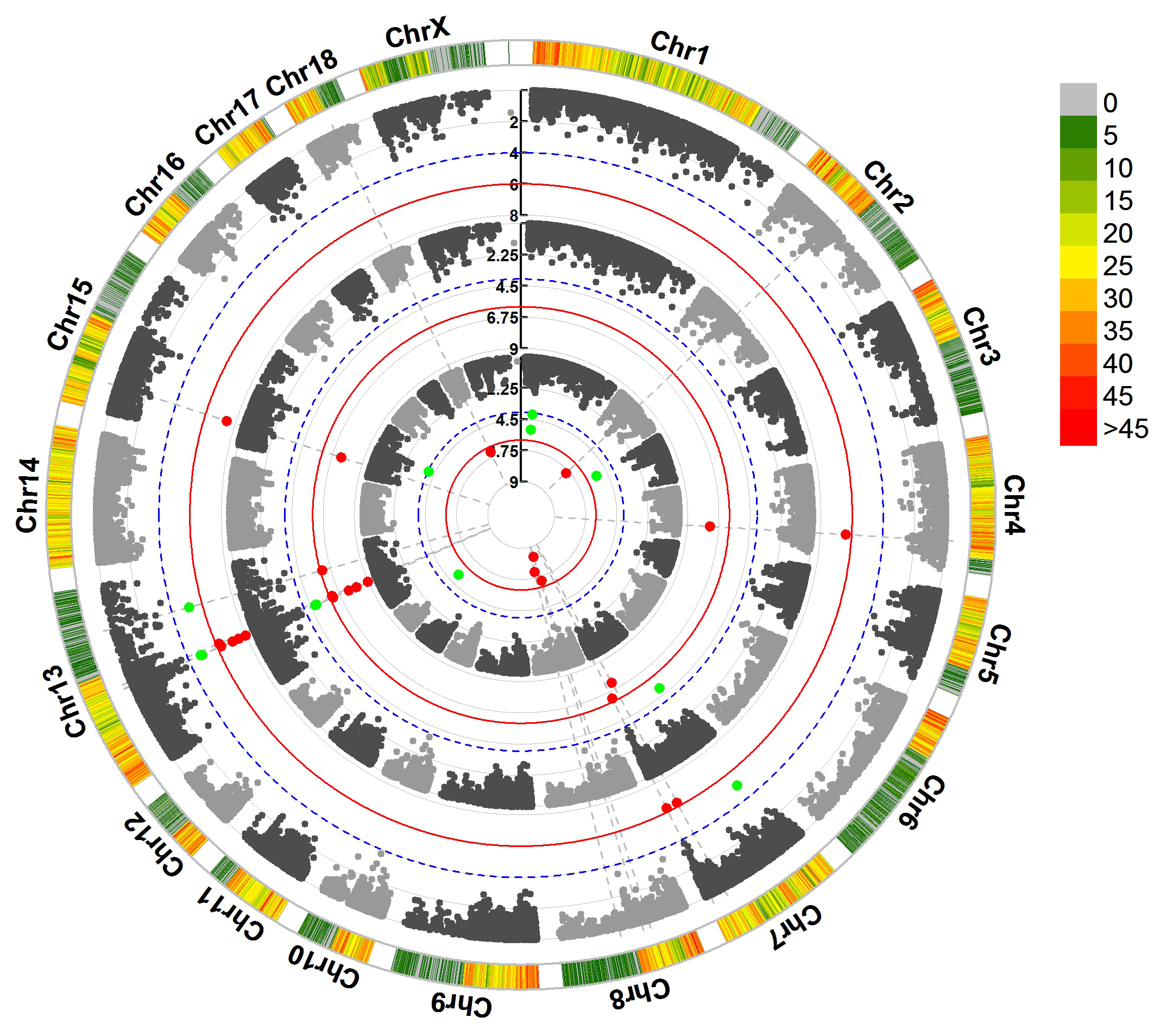


1. **Manhattan plot in rectangular manner for multiple-group GWAS results**

***MVP.Report*** *(pig60K, plot.type="m", multracks=TRUE, threshold=c(1e-6,1e-4), threshold.lty=c(1,2), threshold.lwd=c(1,1), threshold.col=c("black","grey"), amplify=TRUE, bin.size=1e6, chr.den.col=c("darkgreen", "yellow", "red"), signal.col=c("red","green"), signal.cex=c(1,1), file="jpg", memo="", dpi=300)*


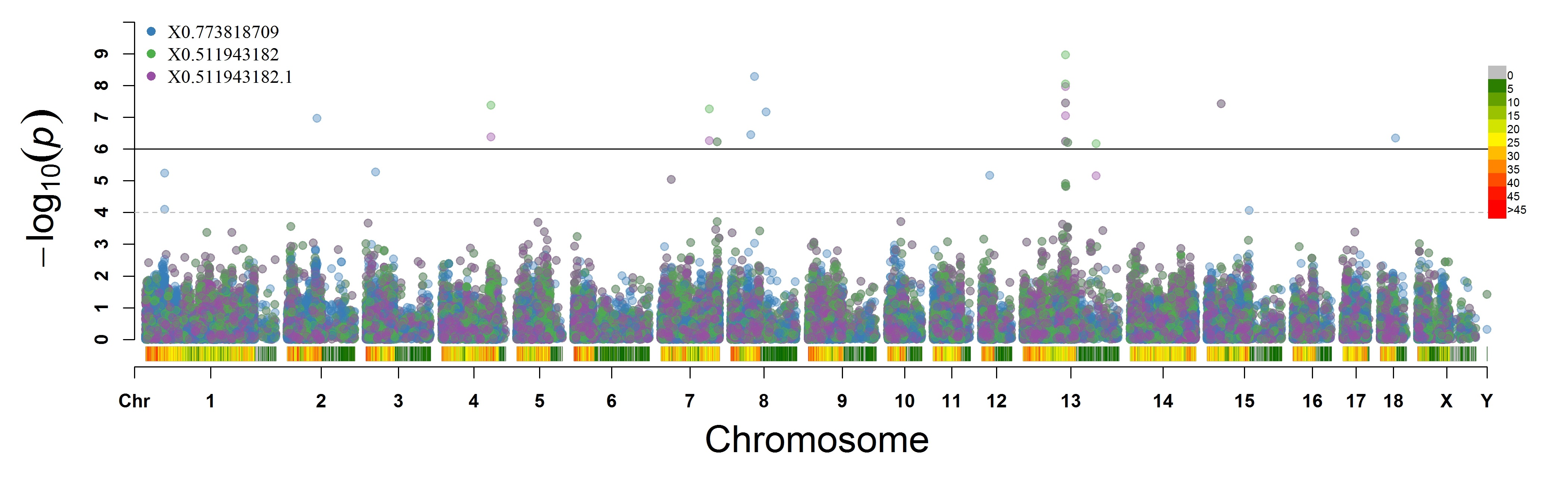


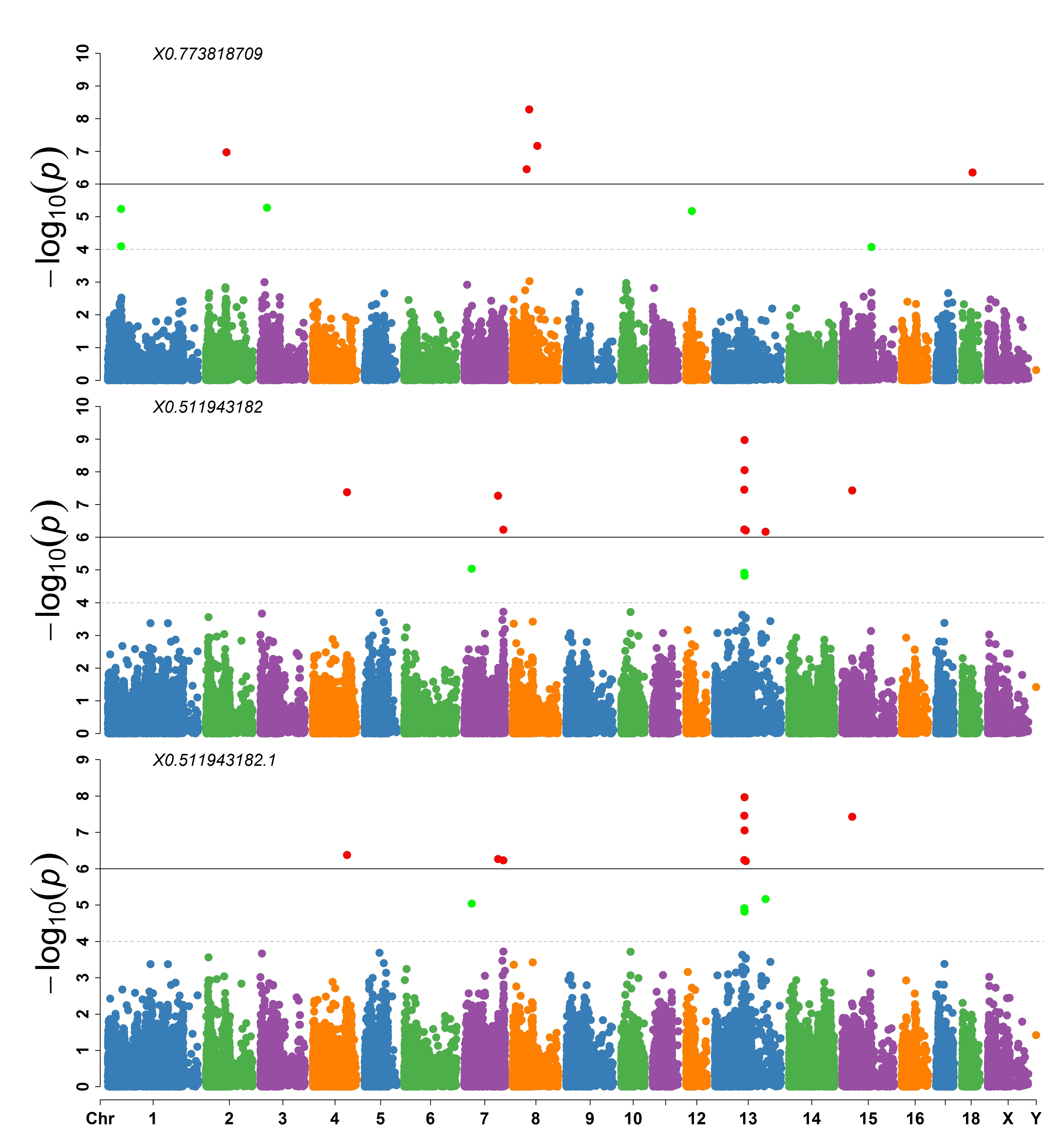


1. **QQ-Plot for multiple-group GWAS results**

***MVP.Report****(imMVP, plot.type="q", col=c("dodgerblue1", "olivedrab3", "darkgoldenrod1"), threshold=1e6, signal.pch=19, signal.cex=1.5, signal.col="red", conf.int.col="grey", box=FALSE, multracks=TRUE, file="jpg", memo="", dpi=300)*


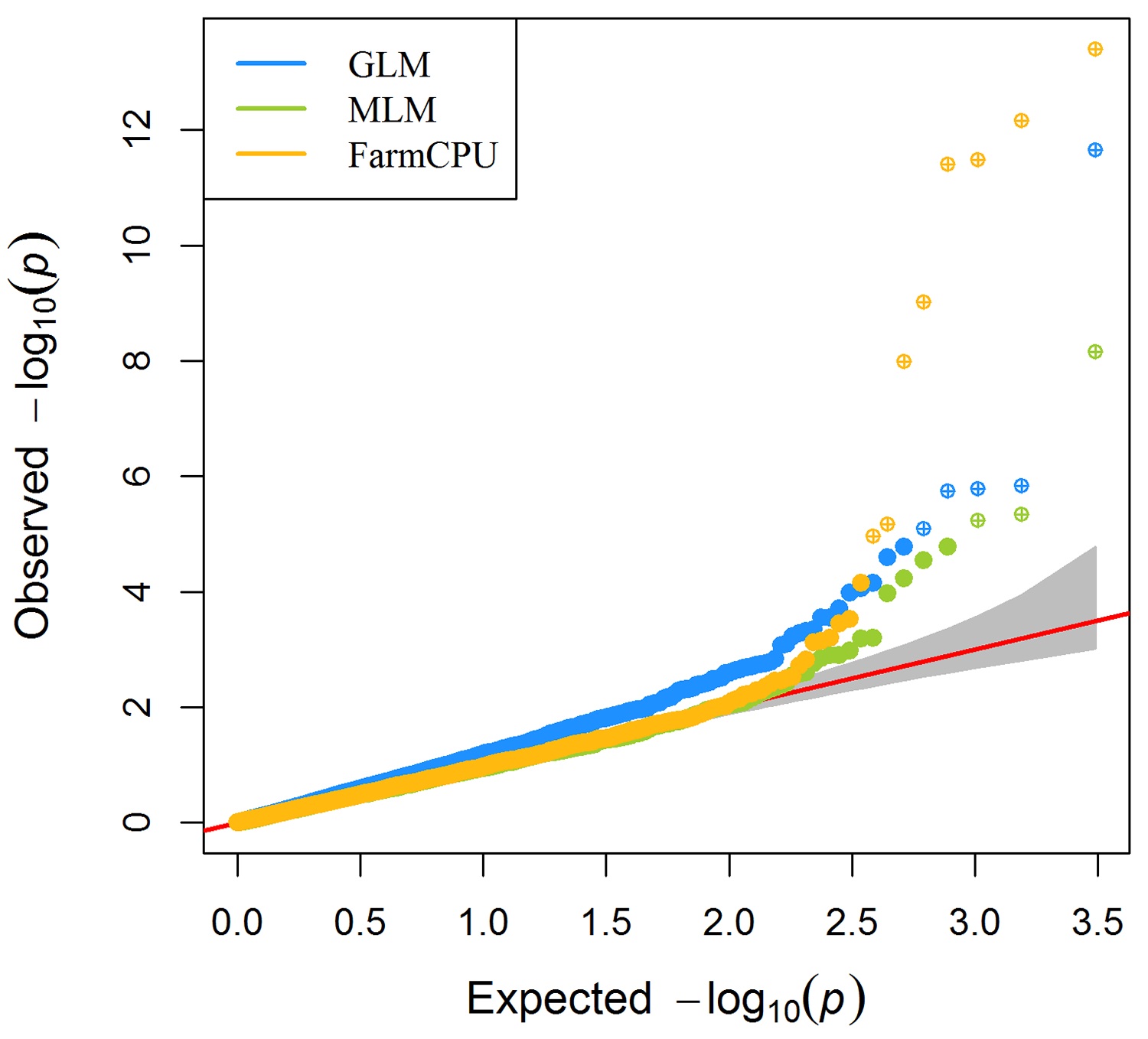

Supplement: Supplementary File S1 — Demo scripts and figures for visualization in rMVP. [file mmc1.docx]
