# Supplementary figures and images for: rMVP: A Memory-efficient, Visualization-enhanced, and Parallel-accelerated Tool for Genome-wide Association Study
Source: Genomics Proteomics Bioinformatics. 2021 Mar 2;19(4):619–28. doi: 10.1016/j.gpb.2020.10.007 (PMC9040015; doi:10.1016/j.gpb.2020.10.007)

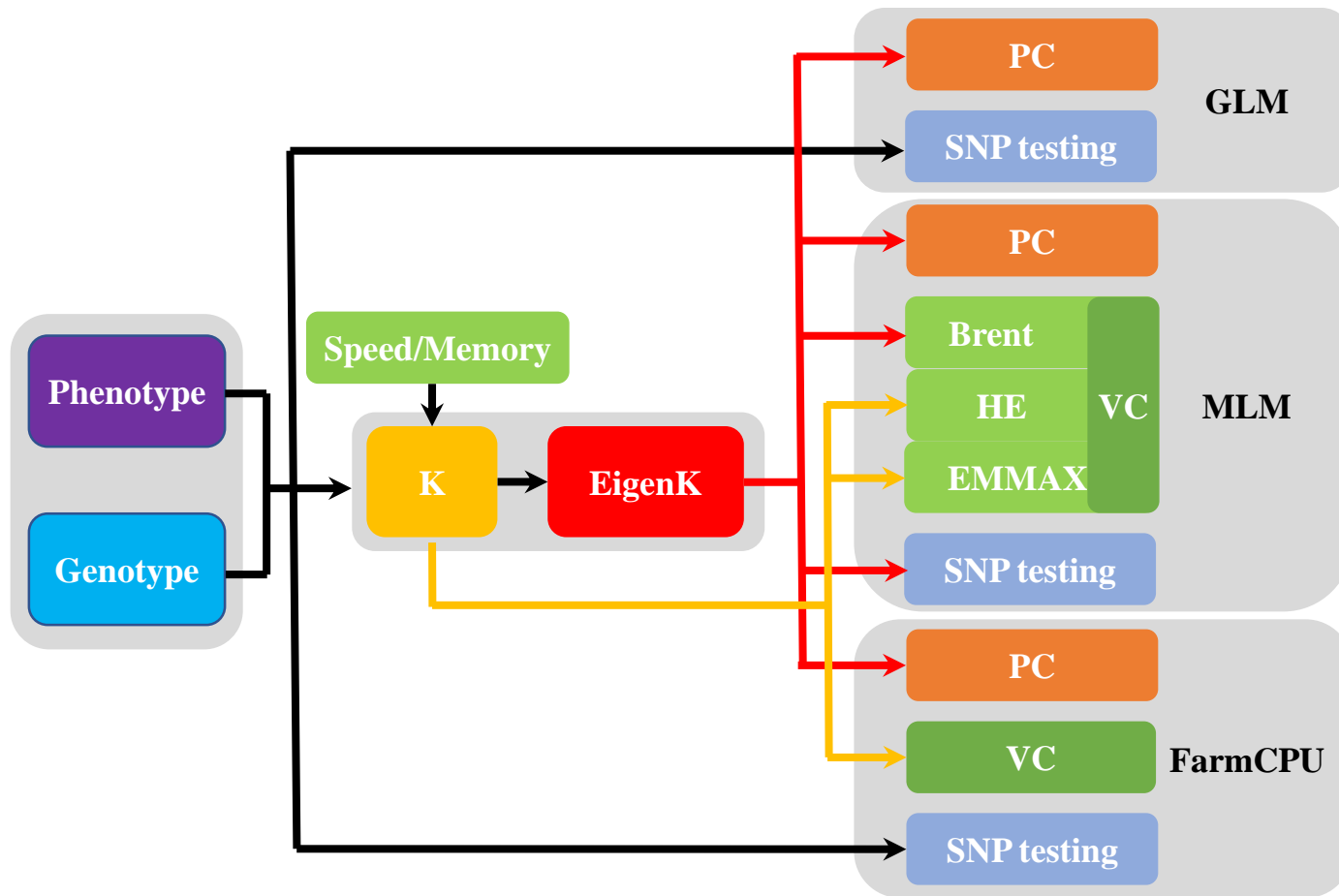

Supplement: Supplementary Figure S1 — The road mapping of whole GWAS procedures in rMVP. K is the Kinship matrix, also known as GRM. EigenK represents the Eigen decomposition of GRM. GRM, genomic relationship matrix; PC, principal component. VC, variance component. [file mmc2.pdf]

**GLM**

**rMVP vs PLINK v2.0**

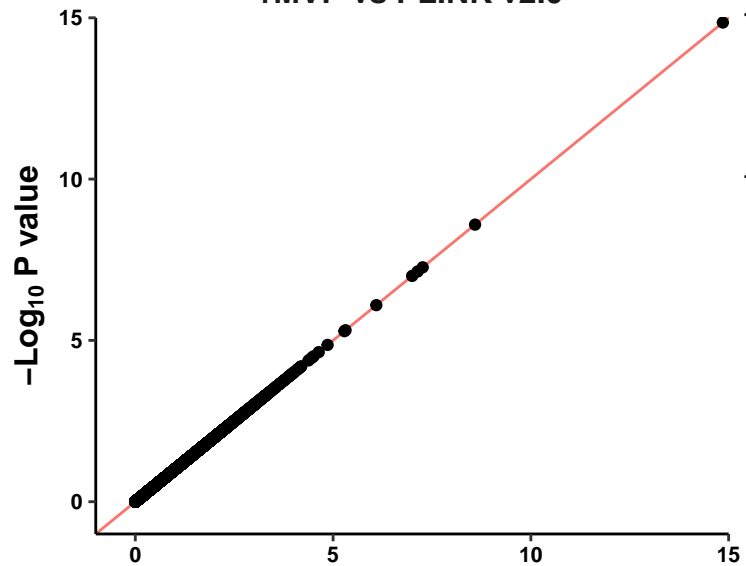

**MLM**

**rMVP vs GEMMA**

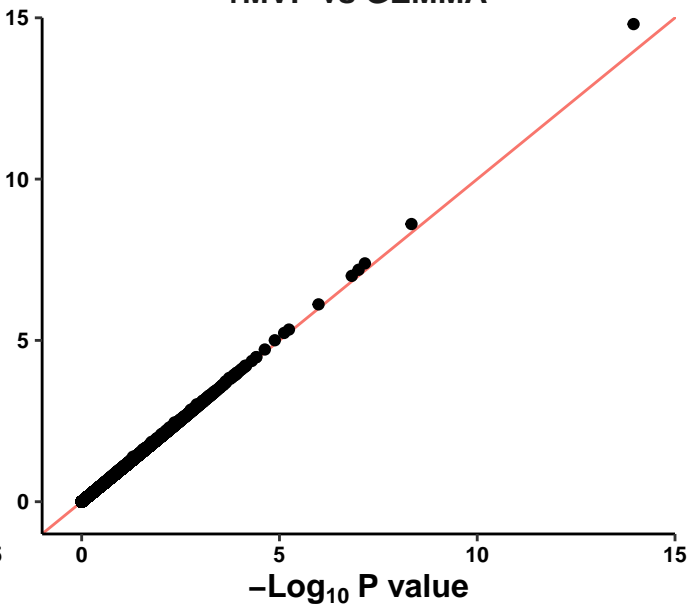

**FarmCPU**

**rMVP vs FarmCPU\_pkg**

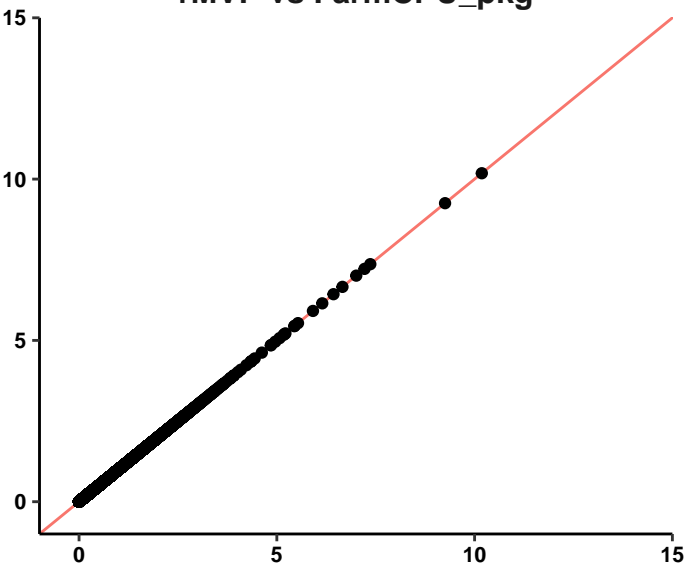

Supplement: Supplementary Figure S2 — Comparison of association results for three GWAS models between rMVP and related software. The experiment was performed on the simulated 16 data units (16,000 samples and 1,600,000 SNPs). The x-axis represents the computed P value in −Log10 format of rMVP for three GWAS models, while the y-axis represents the computed P value in −Log10 format of related software for corresponding GWAS model. [file mmc3.pdf]

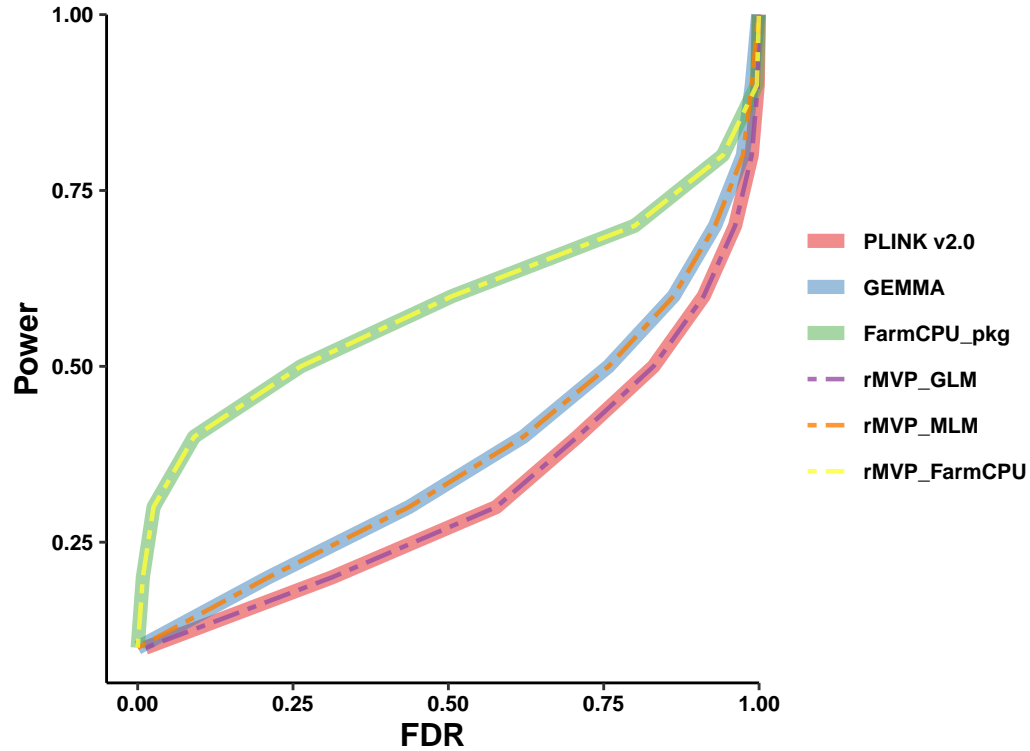

Supplement: Supplementary Figure S3 — Comparison of power and FDR for three GWAS models between rMVP and related software. The experiment was performed using an Arabidopsis dataset, which includes 1178 individuals and 208,794 SNPs. The phenotype was simulated by randomly selected 10 quantitative trait nucleotides (QTNs) following a normal distribution with mean = 0 and variance = 0.1, and the heritability was 0.5. The final results were the average of 100 replicates. FDR, false discovery rate. [file mmc4.pdf]
